# Swimming Exercise and Transient Food Deprivation in *Caenorhabditis elegans* Promote Mitochondrial Maintenance and Protect Against Chemical-Induced Mitotoxicity

**DOI:** 10.1038/s41598-018-26552-9

**Published:** 2018-05-29

**Authors:** Jessica H. Hartman, Latasha L. Smith, Kacy L. Gordon, Ricardo Laranjeiro, Monica Driscoll, David R. Sherwood, Joel N. Meyer

**Affiliations:** 1 Nicholas School of the Environment, Duke University, Durham, NC United States; 2 Department of Pharmacology and Cancer Biology, Duke University, Durham, NC United States; 3 Department of Biology, Duke University, Durham, NC United States; 4 Department of Molecular Biology and Biochemistry, Nelson Biological Laboratories, Rutgers, The State University of New Jersey, Piscataway, NJ United States

## Abstract

Exercise and caloric restriction improve health, including reducing risk of cardiovascular disease, neurological disease, and cancer. However, molecular mechanisms underlying these protections are poorly understood, partly due to the cost and time investment of mammalian long-term diet and exercise intervention studies. We subjected *Caenorhabditis elegans* nematodes to a 6-day, twice daily swimming exercise regimen, during which time the animals also experienced brief, transient food deprivation. Accordingly, we included a non-exercise group with the same transient food deprivation, a non-exercise control with *ad libitum* access to food, and a group that exercised in food-containing medium. Following these regimens, we assessed mitochondrial health and sensitivity to mitochondrial toxicants. Exercise protected against age-related decline in mitochondrial morphology in body-wall muscle. Food deprivation increased organismal basal respiration; however, exercise was the sole intervention that increased spare respiratory capacity and proton leak. We observed increased lifespan in exercised animals compared to both control and transiently food-deprived nematodes. Finally, exercised animals (and to a lesser extent, transiently food-deprived animals) were markedly protected against lethality from acute exposures to the mitotoxicants rotenone and arsenic. Thus, swimming exercise and brief food deprivation provide effective intervention in *C. elegans*, protecting from age-associated mitochondrial decline and providing resistance to mitotoxicant exposures.

## Introduction

A major goal of biomedical research is to not only improve lifespan, but to improve the number of healthy years, or healthspan. It is estimated that behavioral or “lifestyle” choices contribute up to 50% to health outcomes^1^. An accessible, inexpensive, and effective form of lifestyle intervention is physical exercise^2^. Exercise helps maintain good health and reduces the risk of developing chronic diseases including cardiovascular disease, metabolic syndrome, type 2 diabetes, neurological disease, and cancer^2–4^. However, the cellular mechanisms by which exercise confers these systemic protections are not well understood.

One reason that the mechanisms of exercise protection have not been fully characterized is the cost and time investment of long-term exercise intervention required for rodent models or human studies. *Caenorhabditis elegans* nematodes are a promising new model for studying whole-organism effects of exercise intervention throughout an entire lifetime. Further, *C. elegans* represent a unique and powerful tool for mechanistic studies due to their short lifespan, transparent body that facilitates subcellular, cellular, and tissue live imaging, and the many genetic tools that allow molecular pathways to be characterized. Laranjeiro *et al*. recently showed that a single bout of swimming exercise in *C. elegans* induces hallmark features of mammalian exercise, including muscle fat depletion, post-swim locomotory fatigue, oxidation of the mitochondrial matrix in muscle cells, and transcriptional changes in antioxidant genes^5^. The impacts of long-term conditioning with this swimming protocol on *C. elegans* have not yet been reported.

Here we address consequences of longer-term exercise training with an emphasis on mitochondrial health and potential resilience to environmental toxicant challenge. We tested the benefits of exercise to *C. elegans* mitochondria by administering six days of twice-daily swim sessions followed by assessment of mitochondrial health one and five days following cessation of exercise. During each swim session, the animals underwent a brief, transient food deprivation. Therefore, we included a non-exercise group that was subjected to an identical bout of transient food deprivation (tFD) and a control with *ad libitum* access to food, which allows comparison to normally cultured nematodes. We chose mitochondrial endpoints because long-term exercise conditioning in humans and rodents has been demonstrated to increase mitochondrial content and function in skeletal muscle^6,7^, brain^8–10^, and liver^11^. We qualitatively scored mitochondrial morphology in *C. elegans* body wall muscle and measured whole-animal mtDNA content, DNA damage, and respiration as metrics of muscular and organismal mitochondrial health, respectively. Further, given that pollutants are increasingly recognized as playing a role in aging^12^, and globally, pollution-induced disease currently causes about 9 million premature deaths annually (16% of the total^13^), there is growing concern regarding the potential impact of drugs^14–16^, pollution^14,17–20^, and other environmental stressors on mitochondria. Therefore, we also tested the sensitivity of nematodes to the mitochondrial toxicants rotenone and arsenic. Finally, we assayed the lifespan and autofluorescence (as a surrogate marker of healthspan^21,22^) in exercised, tFD, and control animals. Upon discovering phenotypes that appeared to be driven primarily by either tFD or exercise, we tested an additional manipulation of nematode swimming exercise in food-containing media to further disentangle the effects of exercise and food deprivation. Our overall objective was to assess the mitochondrial consequences of exercise and tFD in *C. elegans* in order to establish *C. elegans* as a new model for studying molecular mechanisms underlying exercise-induced benefits to organismal health.

## Results

### A swimming-based exercise regimen for young reproductive adults

**Figure 1:**
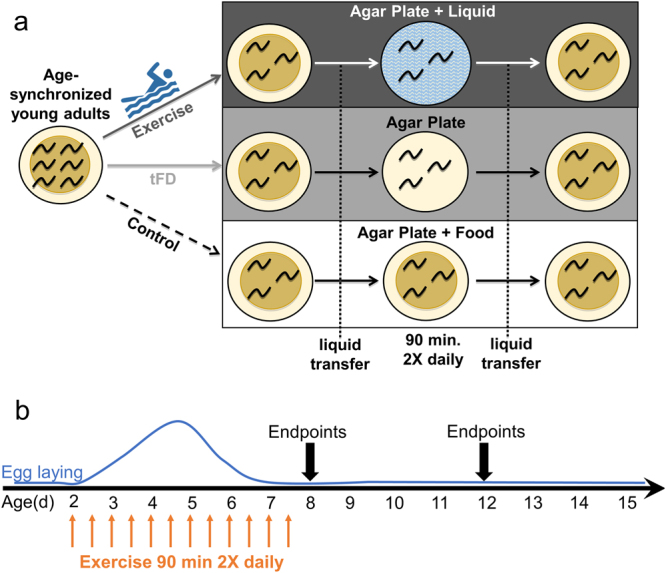
Swimming exercise overview. Panel a, stylized depiction of exercise conditioning protocol. Cartoon depiction of swimming man obtained under Creative Commons CC0 from pixabay.com. For experiments, synchronized young adult N2 nematodes from a single egg preparation were divided into control, transient food deprivation (tFD), and exercise groups and maintained on K-agar plates seeded with OP50 *E. coli* bacteria at 20 °C. Twice daily, animals were transferred to unseeded plates (tFD and swim exercise groups) or to plates seeded with OP50 bacteria (control) for 90 minutes before transferring back to seeded food plates. Panel b, timeline of exercise conditioning and endpoint measurements. Twice daily exercise timepoints are depicted as orange arrows and overlaid with an approximation of the N2 hermaphrodite egg laying schedule shown with a solid blue line to highlight that exercise occurred during the entire adult reproductive period of an unmated animal. Although males should be few, we cannot rule out the presence of males and longer reproductive periods in mated hermaphrodites. See Methods for further details. Endpoints were taken on day 8, one day following cessation of exercise, and on day 12.

We carried out swim exercise conditioning from the young adult stage (48 hours post-transfer of L1 larvae to seeded plates at 20 degrees C; designated “day 2”), twice daily for six days (ending on day 7). For the study, animals were washed off food plates in K-medium and either plated onto unseeded food-free plates inundated with liquid to induce swimming exercise (“exercise” group), onto unseeded food-free plates (“transient food deprivation” or “tFD” group, which features physical manipulations and food availability matched to exercise), or back onto food plates (“control” group, mechanical manipulations implemented, always have *ad libitum* access to food) (Fig. 1a). After six days of the twice-daily swimming exercise regimen, we measured endpoints on day 8 (one day after exercise cessation) and day 12 (five days after ceasing exercise) (Fig. 1b).

### Age-related decline in body wall muscle mitochondrial morphology is improved by exercise

**Figure 2:**
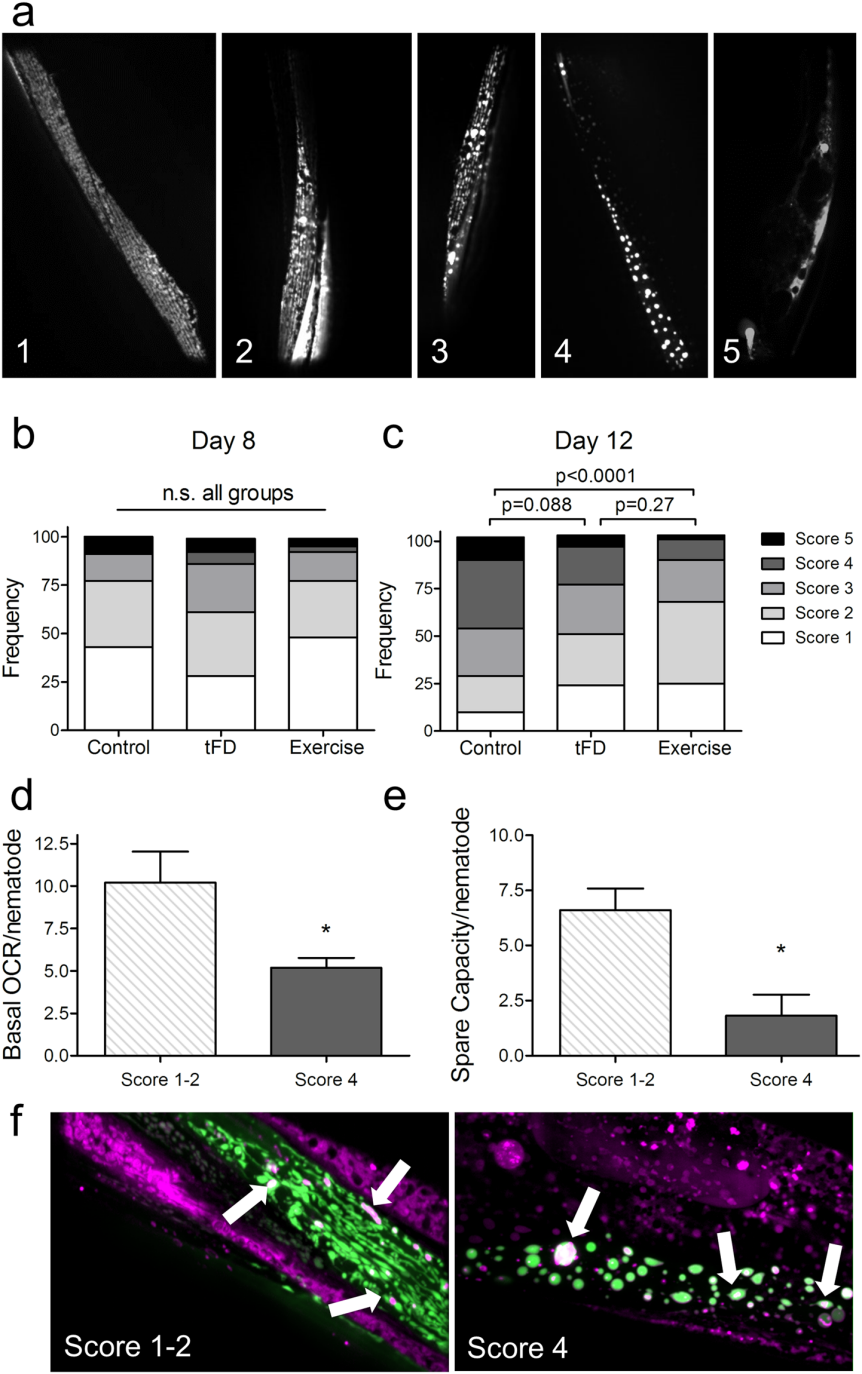
Exercise protects from aging-induced deterioration of body wall muscle mitochondrial morphology. Panel a, representative images demonstrating the scoring system used to visually discriminate different morphologies. Images were taken in the distal posterior (tail region) of the animal to maximally avoid bright autofluorescence of the gut. Score 1 represents highly abundant, highly networked mitochondria with little or no blebbed appearance, while score 2 has highly abundant, networked mitochondria with some blebbing. Score 3 has less abundant mitochondria with gaps in the network and more blebbing, and score 4 has very sparse, enlarged mitochondria. Finally, score 5 was a severe phenotype in which no muscle GFP could be detected (shown in the image is autofluorescence in the tail region). Panel b represents score distributions on day 8 and Panel c scores on day 12. The Y-axis represents the frequency (proportion of 100%) for each score. Images were loaded into a blinding scoring software written in-house for this purpose (see Methods). Data shown represent three individual biological replicates, each with 10–25 animals per group. Global statistical difference among the three distributions was determined using a Chi-squared test, followed by pairwise comparisons using a significance level of 0.017 (Bonferroni adjustment of p < 0.05 for multiple comparisons). Further experiments were performed to test the relative health of mitochondria in animals with high or low scores. First, whole animal respiration was tested using the 24-well Seahorse plates with 25 animals per well. Panel d, basal respiration and Panel e, spare capacity reflect the average oxygen consumption rates from four individual wells. In Panel f, the relative membrane potential was assessed by staining with Mitotracker Red, a cationic dye requiring membrane potential for accumulation in mitochondria. White arrows highlight examples of mitochondria with co-localized GFP and Mitotracker Red in both networked and fragmented mitochondrial phenotypes.

Mitochondrial adaptations in muscle are a hallmark of exercise training^7^ and dietary restriction^23^; therefore, we assessed muscle mitochondrial morphology using a strain that expresses mitochondrial matrix-targeted GFP in body wall muscle (SJ4103). We focused our assessment of muscle mitochondrial morphology by imaging in the tail region, which features minimal interference from gut autofluorescence and in which mitochondrial morphology may decline faster than the anterior region as the animals age (unpublished observations). Decline in muscle mitochondria occurs during mammalian and *C. elegans* aging^24,25^, and in the presence of some mitochondrial toxicants^26,27^. This decline is characterized by mitochondrial networks becoming less organized, more sparse (fewer mitochondria) and by accumulation of large dysfunctional mitochondria^24,28^. We evaluated mitochondrial images blind to exercise regimen, scoring according to a qualitative scale ranging from 1 (healthy: highly networked, robust, abundant mitochondria) to 4 (degenerated: sparse, enlarged, fragmented mitochondrial networks); we awarded an additional score of 5 when GFP-tagged mitochondria were undetectable in the tail body wall muscle (Fig. 2a).

We compared control, tFD, and exercise animals one day after training was complete at day 8, and five days after on day 12. Overall, the three groups had similar mitochondrial network profiles on day 8 (Fig. 2b, p = 0.056, chi-squared test). On day 12, we recorded a definitive change in body wall muscle morphology in all groups (Fig. 2c, p < 0.0001, chi-squared test for global differences). The control group displayed the largest decline in the frequency of score 1 (healthy mitochondria) animals and a large increase in scores 4 and 5 (highly fragmented/sparse mitochondria and absent mitochondria, respectively). By contrast, the exercise group exhibited significantly better-maintained mitochondria later into life. For example, whereas only ~24% of sedentary control animals scored as the healthy youthful scores of 1 and 2, ~60% of exercised animals scored as 1–2 morphologies. The exercise group was significantly different from the control, but not different from tFD, which was intermediate between the other two groups.

To more directly assess how scores of 1–2 functionally compared to worse scores such as 4, we performed whole-animal oxygen consumption (respiration) analysis on 12-day old control animals from each phenotype, finding that both basal respiration (Fig. 2d) and spare capacity (Fig. 2e) were higher in the animals with more networked mitochondrial morphologies (scores 1–2). However, mitochondria from both morphological categories accumulated the cationic mitochondrial dye MitoTracker Red (Fig. 2f), which relies on membrane potential for uptake. This indicates that even sparse and fragmented mitochondria do retain some membrane potential, although our technique does not permit quantification. However, we also noted that whole-animal staining of mitochondria with MitoTracker Red was more sparse in animals with a poor score of 4 in muscle mitochondria compared to the score of 1–2. Overall, mitochondrial morphology becomes significantly more fragmented and globular in control post-reproductive adult nematodes, mitochondrial function measured as basal and maximal oxygen consumption declines, and there is an intervention-specific impact on old-age mitochondrial biology.

### Nuclear DNA content and genome integrity change with age, but this progression is not markedly changed by swim experience

**Figure 3:**
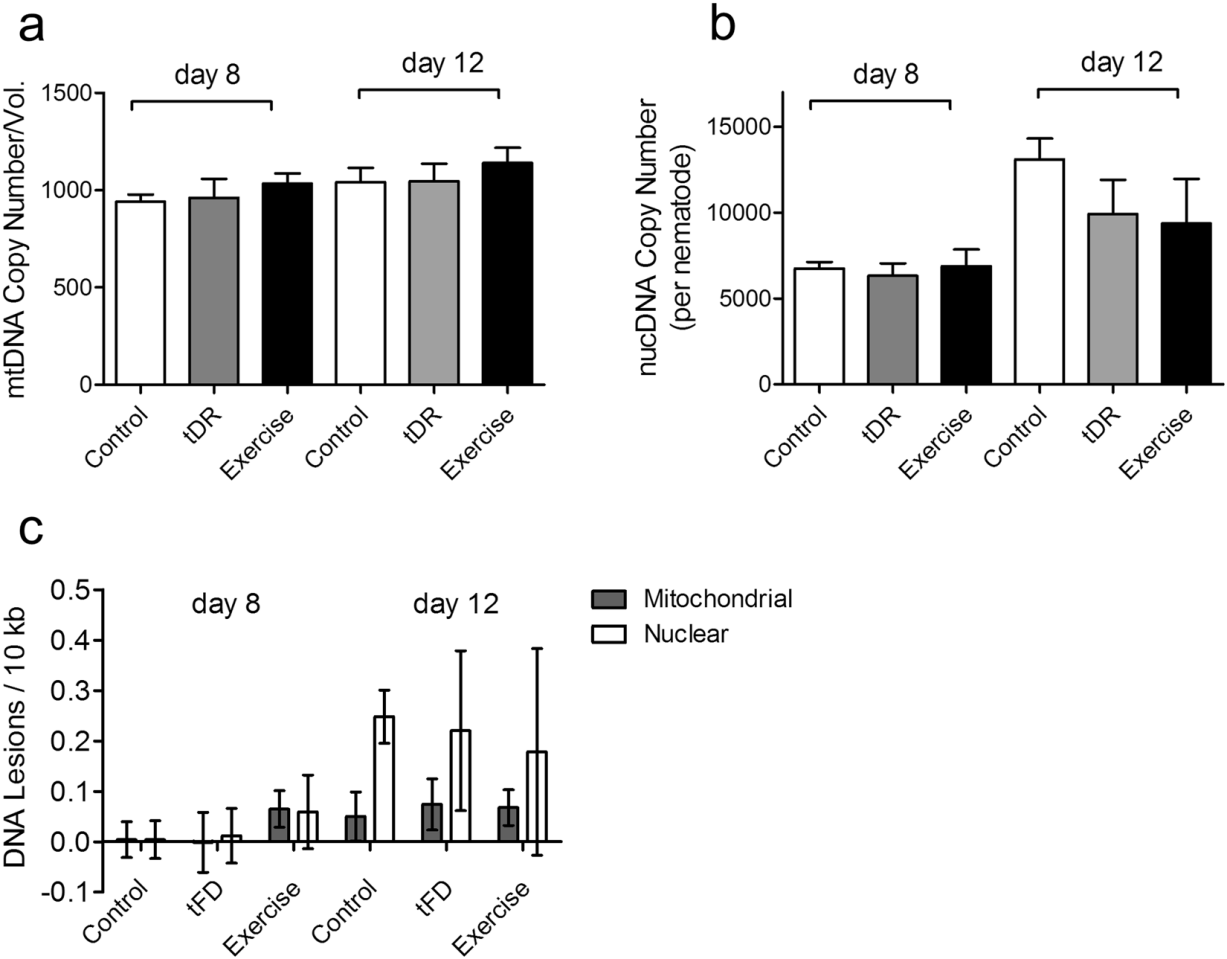
Exercise does not change mitochondrial or nuclear DNA copy number, nor does exercise reduce DNA damage. Panel a, mitochondrial DNA copy number and Panel b, nuclear DNA copy number, determined as previously described^29^. Panel A y-axis represents relative copy number (absolute mitochondrial DNA copy number divided by worm volume) due to size differences between groups, while Panel B y-axis represents absolute nuclear DNA copy number due to all animals having a fixed number of cells. There were no significant differences between treatment groups at either time point, as determined by one-way ANOVA. Panel c, DNA lesions in mitochondrial and nuclear genomes were determined by long QPCR amplification as described^29^. Day 8 control animals were considered to be baseline lesion levels in the analysis; thus, all lesion scores are relative to lesions in the control animals. Data shown are the average of three technical replicates (each from a pooled sample of 6 worms) and two biological replicates. Error bars represent the standard error of the mean.

Given that mitochondrial organization in body wall muscle was physically improved after exercise, we addressed whether mitochondrial content might also increase as a diet/exercise adaptation, using mitochondrial DNA copy number as a measure. We determined mitochondrial and nuclear DNA copy number using real-time PCR amplification of a mitochondrial-encoded gene (*nduo-1*) and a nuclear-encoded gene (*cox-4*), as previously described^29^. Overall, after normalizing to animal volume, which changes with experimental manipulations (see Supplementary Fig. S1), we found no significant differences in mitochondrial DNA copy number between day 8 and day 12, and no difference among control, tFD, or exercised worms (Fig. 3).

In contrast, we found that the nuclear DNA copy number per nematode increased between day 8 and day 12 (Fig. 3b), as previously observed^30^; however, we measured no difference between control, tFD, and exercised groups. We also analyzed DNA integrity in the mitochondrial and nuclear genome using the long qPCR assay^29^. Although basal nuclear (but not mitochondrial) DNA lesions increased between day 8 and day 12 when we combined and compared all treatment groups (p = 0.0392, Student’s paired t-test), we found no significant differences among the three groups on either day. Our data support increased basal DNA damage or an overall decline in repair of basal DNA damage with age, which on average is not changed by tFD or exercise.

### Whole-nematode mitochondrial respiration is altered by transient food deprivation and exercise conditioning

**Figure 4:**
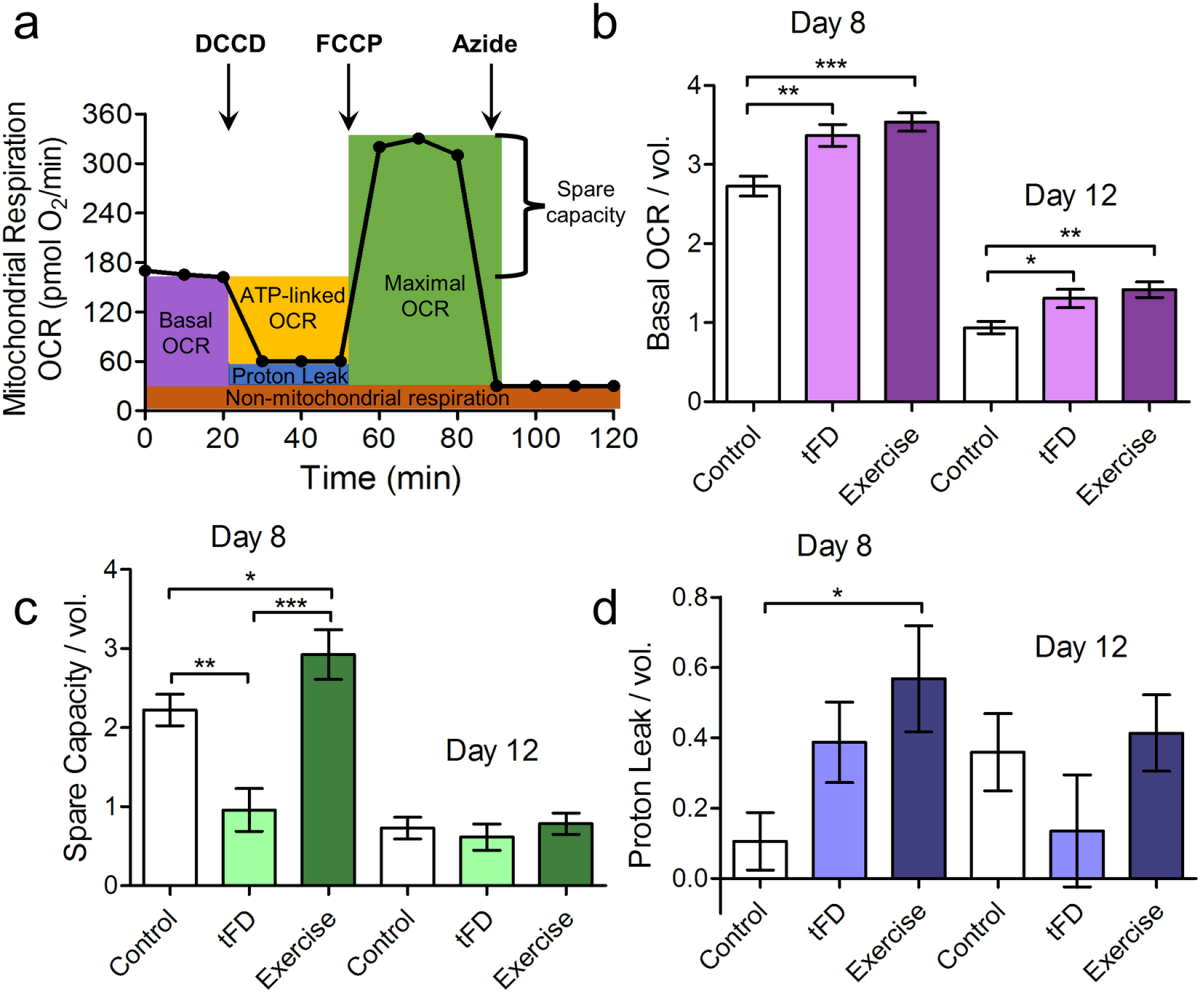
Exercise and transient food deprivation impact mitochondrial basal respiration, spare capacity, and proton leak. For experiments, nematodes were washed off plates, rinsed, and allowed to clear their guts for 20 minutes. The animals were rinsed once more and then loaded into 24-well Seahorse plates, 25 nematodes per well, for measurement of oxygen consumption rate (OCR). All OCR measurements are normalized per nematode and per average nematode volume due to size differences between groups (Supplemental Figure S1). Final units are pmol O_2_/min/nematode/µm^3^ × 10^3^. Basal OCR was measured along with OCR following injection of FCCP, DCCD, or sodium azide (Panel a). These measurements were then normalized per nematode and per volume and used to calculate the following parameters: Panel b, basal respiration, Panel c, spare capacity (FCCP response – basal OCR), and Panel d, proton leak (DCCD response – sodium azide response). Statistical significance was assessed by a two-way ANOVA followed by a Bonferroni posttest comparing all groups to each other. Data represents the mean and standard error from 3 biological replicates. Asterisks represent statistical significance: *p < 0.05; **p < 0.01; ***p < 0.0001).

After finding that organismal mitochondrial DNA content was unchanged with exercise, we addressed whether mitochondrial function, rather than number, might be modified consequent to food deprivation and exercise. To test this hypothesis, we undertook functional analysis of mitochondrial respiration in whole nematodes using measurement of oxygen consumption rates with a Seahorse XFe24 Extracellular Flux Analyzer (assay summarized in Fig. 4a). Basal oxygen consumption, which reflects baseline mitochondrial activity in nematodes (Fig. 4b), was higher in both exercise and tFD groups compared to the control group (p < 0.05, Tukey’s Multiple Comparison Test). Our findings indicate that basal oxygen consumption rate (OCR) changes cannot be attributed to the swim exercise *per se*. Rather, daily transient food deprivation might stimulate basal mitochondrial activity for these nematodes. Interestingly, we find that ATP-linked respiration is unchanged among the groups (Supplementary Fig. S2).

By contrast, spare capacity (FCCP uncoupled maximal rate – Basal OCR, Fig. 4a, green shading), which is the ability of the mitochondria to respond to a higher demand for energy, was specifically changed by exercise. Spare capacity (Fig. 4c) differed between the groups soon after cessation of training on day 8 (p < 0.0001, one-way ANOVA), but this difference was not maintained at 5 days post-exercise. The dramatic increase in spare capacity for day 8 animals was unique to exercise, suggesting that exercise conditioning may enhance this aspect of mitochondrial function to enable animals to respond to higher-energy demands when necessary. It is also possible that exercise changes other factors affecting maximal oxygen consumption (including substrate availability), which may contribute to the observed differences in spare capacity.

Swim exercise also trended to support enhanced proton leak (DCCD-inhibited OCR – Azide-inhibited OCR, Fig. 4d), which could reflect more fused mitochondria or an adaptation that might limit reactive oxygen species production. Overall, our data revealed functional differences in mitochondria at the whole-nematode level, suggesting an organismal phenotype of increased mitochondrial health after transient food deprivation and exercise conditioning.

### Exercised animals are protected from sodium arsenite-induced lethality

**Figure 5:**
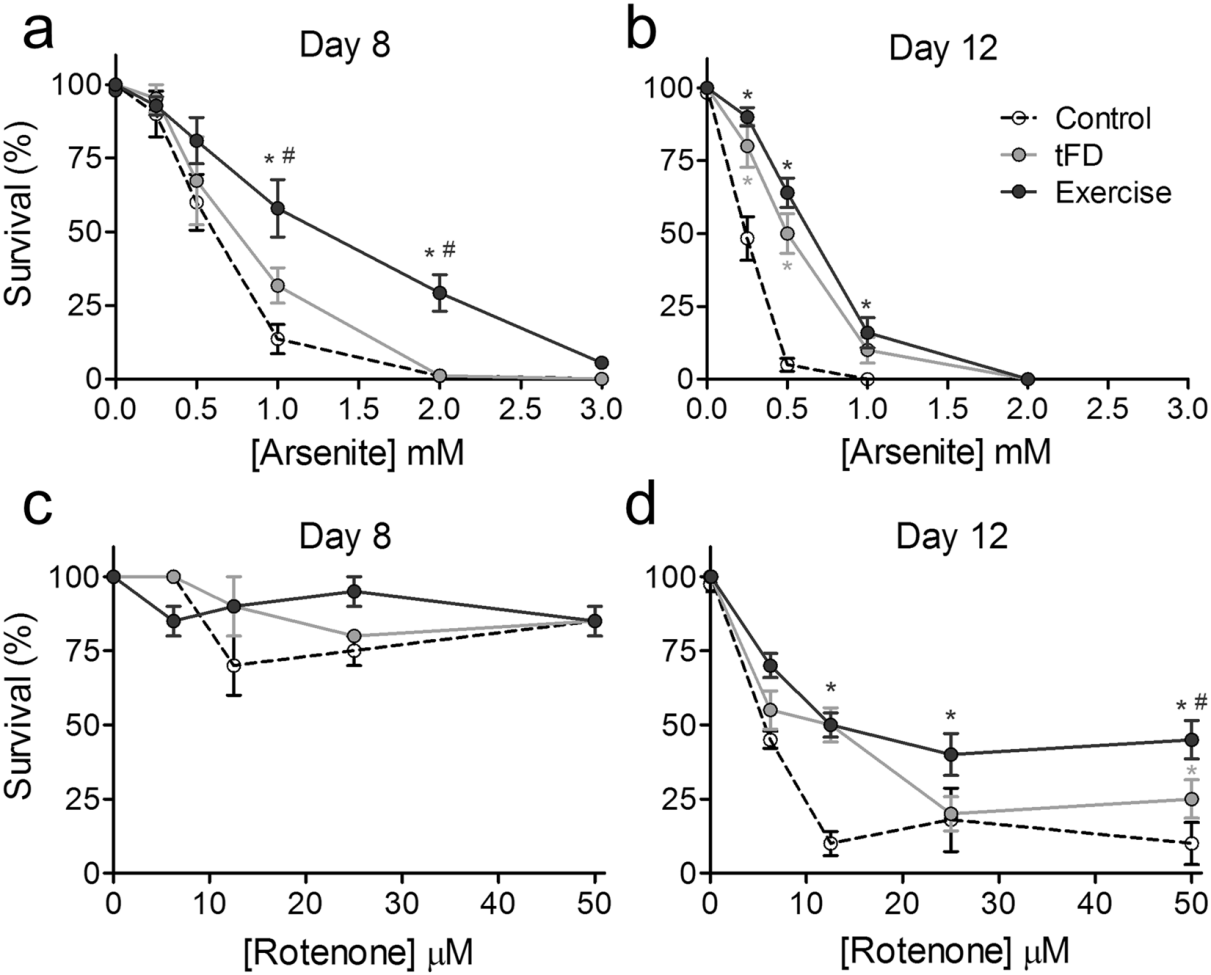
Exercise protects against lethality from exposures to arsenite and rotenone. Panels a and b represent 24-hour survival in varying concentrations of arsenite for day 8 and day 12 animals, respectively. Panels c and d are 24-hour survival of day 8 and 12 nematodes, respectively, in rotenone. For experiments, 10 nematodes were picked into a 96-well plate in duplicate wells for each dose. Exposures were carried out for 24 hours, following which survival was assessed by a harsh touch with a platinum wire. Data shown are the average of three biological replicates. For individual traces see Supplementary Information, Figs S3 and S4. Statistical significance was assessed by a two-way ANOVA followed by a Bonferroni posttest comparing all groups to each other. Significance is indicated by an asterisk * if the data are significantly different, p < 0.05, compared to the control group and a symbol ^#^ if the data are significantly different, p < 0.05, compared to the tFD group. For analysis of LC_50_ curves, see Tables 1 and 2.

Both age and relative inactivity may compromise mitochondrial health, limiting the robustness of the organismal response to environmental toxins. For example, although the mechanism of action of arsenite is complex, it involves significant mitochondrial impairment^27,31,32^. Therefore, we tested whether the improved mitochondrial health of exercised animals protected them from lethality after a 24-hour exposure of arsenite. On day 8, arsenite caused a dose-dependent decrease in survival of animals (Fig. 5a, individual traces in Supplementary Fig. S3), with the control group being the most sensitive. When fit to LC_50_ curves, we found that the tFD group had a slightly but not significantly higher LC_50_ compared to control (Table 1). By contrast, the exercise group had a 2.8-fold higher LC_50_, demonstrating significant protection consequent to the swimming regimen. On day 12, all groups were more sensitive to arsenite exposure compared to day 8 (Fig. 5b, individual traces in Supplementary Fig. S3)^27^, but tFD and exercise groups were significantly protected (see also Table 1), again suggesting long-term benefits of limited intermittent food restriction aspect of our protocol. Overall our data reveal that exercise confers resistance to arsenite exposure (consistent with healthier mitochondria), but that specific benefits are more readily apparent 24 hours post training.

**Table 1:** LC_50_ values after 24-hour exposure to sodium arsenite.

Group	LC_50_, mM (standard error)	Significance?	
		vs. Control	vs. tFD
*Day 8*			
Control	0.47 (0.086)	—	—
tFD	0.64 (0.094)	n.s., p = 0.24	—
Exercise	1.3 (0.070)	p < 0.0001	p = 0.0061
*Day 12*			
Control	0.12 (0.032)	—	—
tFD	0.39 (0.10)	p < 0.0001	—
Exercise	0.54 (0.084)	p < 0.0001	n.s., p = 0.19

Animals were subjected to twice-daily 90 min swim exercise beginning at young adult and continuing for 6 days. Lethality was tested on day 8 (the day after ceasing swim exercise) and on day 12. Significance was determined by pairwise comparison of the nonlinear LC_50_ fits using the extra sum-of-squares F test.

### Exercise protects nematodes from rotenone lethality later in life

Rotenone is a potent and specific inhibitor of mitochondrial respiratory Complex I, and rotenone toxicity arises from this inhibition^33^. When we exposed animals to rotenone on day 8 we observed minimal lethality in all groups up to 50 µM (Fig. 5c, individual traces in Supplementary Fig. S4), which is close to the limit of rotenone solubility. However, when we measured rotenone sensitivity on day 12, we observed a dose-dependent decrease in survival (Fig. 5d, individual traces in Supplementary Fig. S4), with the exercise group being two-fold more protected (LC_50_ values in Table 2). Again, although some effects can be associated with transient food deprivation, there is a clear consequence of exercise training that is distinct from tFD, which can be detected even several days after training.

**Table 2:** LC_50_ values after 24-hour exposure to rotenone.

Group	LC_50_, µM (standard error)	Significance?	
		vs. Control	vs. tFD
*Day 8*			
Control	N.D.^a^	—	—
tFD	N.D.	—	—
Exercise	N.D.	—	—
*Day 12*			
Control	3.8 (1.2)	—	—
tFD	9.6 (1.1)	p = 0.0004	—
Exercise	18 (1.2)	p < 0.0001	p = 0.0046

Animals were subjected to twice-daily 90 min swim exercise beginning at young adult and continuing for 6 days. Lethality was tested on day 8 (the day after ceasing swim exercise) and on day 12. Significance was determined by pairwise comparison of the nonlinear LC_50_ fits using the extra sum-of-squares F test.

^a^Rotenone did not cause significant lethality on day 8 at highest soluble concentrations (see Fig. 4c); therefore, LC_50_ values could not be calculated.

### Exercise conditioning confers positive effects on lifespan

**Figure 6:**
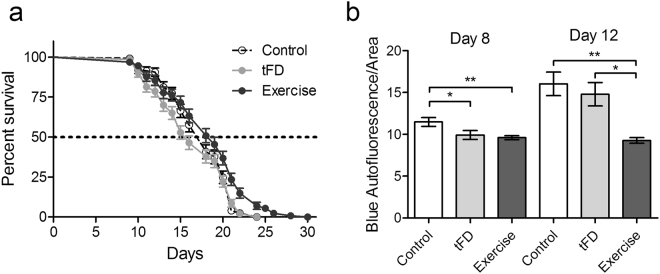
Exercise conditioning extends lifespan and reduces blue autofluorescence “death signal”. Panel a is the compiled lifespan data from three individual biological replicates of n = 50 animals each. For individual traces, see Supplementary Fig. S5. Panel b represents blue autofluorescence, normalized to worm area. Statistical significance was assessed by a one-way ANOVA followed by a Tukey posttest comparing all groups to each other. Autofluorescence data represents the mean and standard error from 3 biological replicates. Asterisks represent statistical significance: *p < 0.05; **p < 0.01).

After testing muscle and organismal mitochondrial health, we also wanted to see if the protection extended to increasing the lifespan or healthspan of the animals. In the lifespan assay, we combined three biological replicates (individual experiments shown in Supplementary Fig. S5), revealing statistically significant but relatively modest differences between the three groups (Fig. 6a, p = 0.0015, Mantel-Cox test), with median lifespans of 18, 16, and 19 days for control, tFD, and exercise, respectively. Pairwise comparison of groups revealed no significant difference between control and tFD (p = 0.37, Mantel-Cox test), potentially due to high variability in the tFD group. However, we observed significant differences between the exercise group as compared to both control (p = 0.0020, Mantel-Cox test) and tFD (p < 0.0001, Mantel-Cox test). Therefore, regular exercise throughout the adult reproductive period was associated with a modest increase in lifespan.

As a complementary approach to the lifespan analysis, we measured a proxy of declining health – autofluorescence, which is reported to increase as nematodes age^21,22^. Blue autofluorescence arises from accumulation of anthranilate and indicates that animals are near death^21,34^. We observed increased blue autofluorescence in both the control and tFD groups on day 12 compared to the exercise group (Fig. 6b), suggesting that as a population, more animals were near death in the control groups compared to the exercise group, which is consistent with the lifespan analysis.

Red autofluorescence also changes with age and is reported to correlate with healthspan^21,22^. We observed complex patterns of red fluorescence change in the control and tFD groups (Supplementary Fig. S6A), but signals from the exercise group were higher than the tFD group on both day 8 and day 12, suggesting differences in post-reproductive exercised animals that may be attributed to the training experience and, contrary to age-related pigment accumulation, might be interpreted as stronger health. Further studies are needed to identify the pigment that fluoresces in the red wavelengths and determine how it could be impacted by food restriction and exercise conditioning.

### Exercise conditioning in food-containing media confers some, but not all, protections observed from exercise in the absence of food

**Figure 7:**
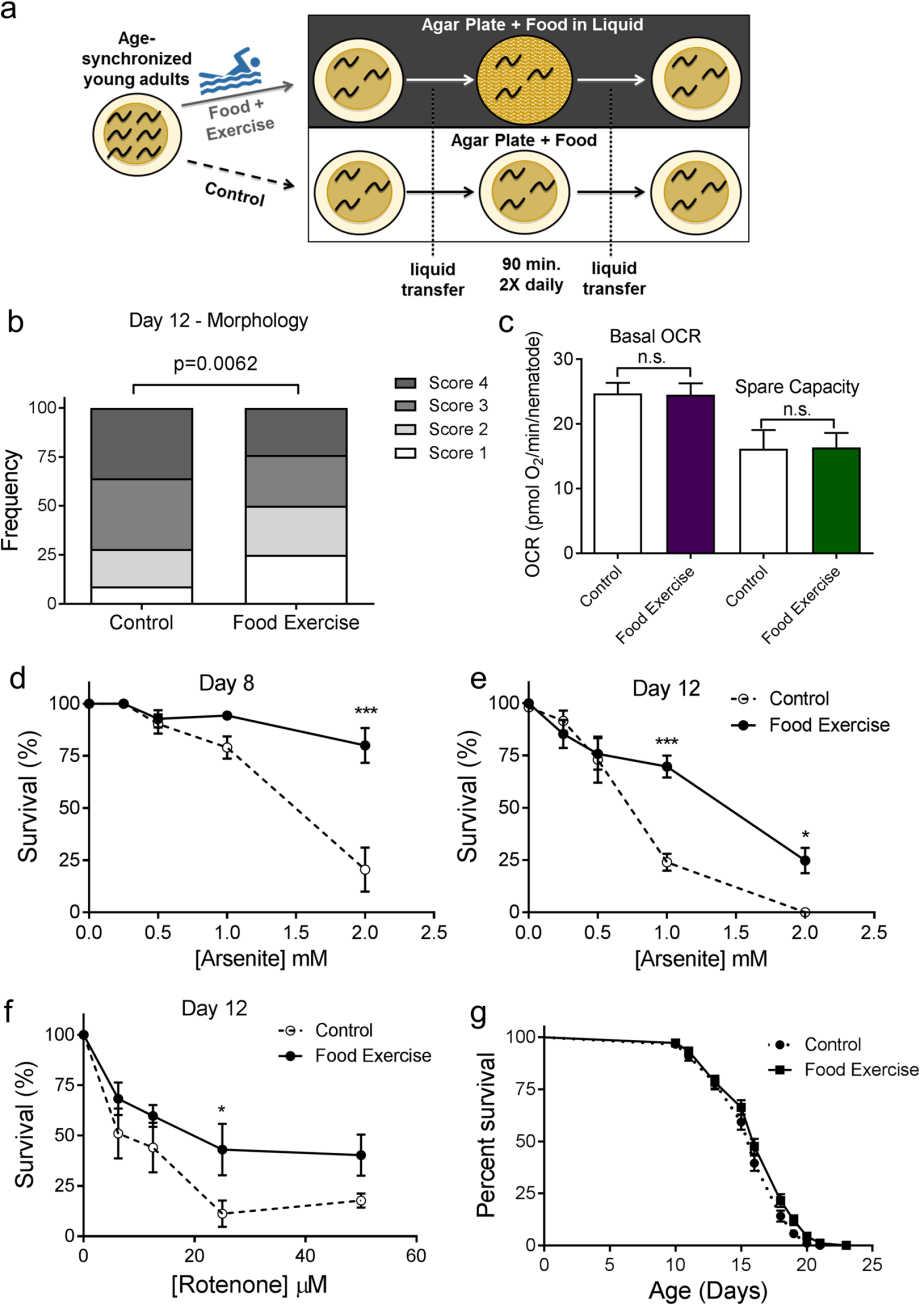
Exercise conditioning in food-containing medium protects muscle mitochondria and ameliorates toxicity of mitotoxicants, but fails to increase respiration or extend lifespan. In Panel a, a stylized depiction of the experimental design is shown; the design is described in detail in Methods. Briefly, animals were subjected to a similar 6-day exercise regimen in media containing OP_50_ bacteria. Cartoon depiction of swimming man obtained under Creative Commons CC0 from pixabay.com. Panel b shows score distributions for mitochondrial morphology on day 12, as described for Fig. 2. Data shown are from n = 70 animals from each group from two independent biological replicates. Statistical significance is based on Chi-squared analysis of the distributions. Panel c represents the respiration in control animals and those exercised in food-containing media, as described for Fig. 4. Data shown are from 10 technical replicates and two independent biological replicates. Panels d and e represent 24-hour survival in varying concentrations of arsenite for day 8 and day 12 animals, respectively. Panel f shows 24-hour survival of day 12 nematodes in rotenone. Survival for rotenone was not performed on day 8 due to high resistance of day 8 adults to rotenone (see Fig. 5). For lethality, data are from two biological replicates and asterisks represent significance in a two-way ANOVA. For corresponding LC_50_ values, see Supplementary Tables S2 and S3. Panel g is the compiled lifespan from two individual biological replicates of n = 100 animals each.

While it was clear that transient food deprivation and exercise resulted in significant protection to age- and chemical-induced mitochondrial damage, we could not tell if these interventions were working through similar or different mechanisms. For example, it is possible that the protections afforded by exercise were simply an effect of increasing energy expenditure while reducing energy intake (i.e. increasing the energy deficit). In an effort to determine whether exercise has effects that are separate from reduction in food intake, we performed the same exercise regimen in liquid media containing food for the animals (OP50 bacteria, Fig. 7a). After swimming exercise in food, nematodes on day 8 were not smaller in size compared to the controls, unlike the transiently food-restricted and exercised animals (Supplementary Fig. S1). However, they maintained significantly healthier mitochondrial networks compared to the controls (Fig. 7b), with ~50% of fed exercised animals having a score of 1 or 2 on day 12 compared to only 25% in the non-exercised controls. This was similar to the protection of muscle mitochondria observed with exercise in liquid without food; therefore, protection of muscle mitochondrial networks is driven mainly by exercise. By contrast, there was no effect of exercise in food on whole-animal basal respiration or spare respiratory capacity (Fig. 7c). Nevertheless, exercise in food protected against arsenite exposure on day 8 (Fig. 7d), resulting in an almost 5-fold increase in the LC_50_ value, and on day 12 (Fig. 7e), resulting in a 2-fold increase in the LC_50_ (Supplementary Table S2). Similarly, exercise in food also resulted in protection from rotenone exposure (Fig. 7f) with LC_50_ values 3-fold higher than controls (Supplementary Table S3). Finally, there was no difference in median lifespan between controls and animals exercised in food-containing media (16 days for each), despite a weak statistical significance between the curves (p = 0.021, Fig. 7g). Together, these findings suggest that for some health outcomes (muscle mitochondrial networks, resistance to chemical exposures), exercise may be working through a different pathway than food restriction, while other outcomes (respiration and lifespan) require food restriction, alone or combined with exercise.

## Discussion

Exercise and dietary restriction are protective against many degenerative effects of aging, and *C. elegans* represents a powerful model organism for identifying the mechanisms underlying systemic benefits of theses interventions. Herein, we show that long-term exercise and transient food deprivation in *C. elegans* are protective against aging-induced changes in mitochondrial morphology in muscle and mitochondrial function (respiration) in whole animals. Furthermore, we show that animals that have undergone this long-term regimen are protected against exposure to the mitochondrial toxicants rotenone and arsenic; such protection is important given the importance of toxicant exposures in aging^12^ and global disease^13^. Finally, we show a modest but significant lifespan extension from swimming exercise in the transient absence of food. These striking muscular and organismal mitochondrial benefits, in conjunction with increased lifespan, demonstrate robust and persistent protection as a result of transient caloric restriction and exercise conditioning.

Sarcopenia, or aging-induced loss of muscle tissue, is preceded by a decline in mitochondrial content and function in rodent^35,36^ and human^28,37^ studies. *C. elegans*, like mammals, undergo an aging-induced loss of locomotor activity that is accompanied by loss of sarcomeres and accumulation of lipid droplets in muscle tissue^25^. We show here that as nematodes age, they undergo a progressive decline in muscle mitochondrial networks. Ultimately, in the most dramatic phenotypes, the muscle mitochondria are enlarged, sparse, and disconnected. This “enlarged mitochondria” phenotype has also been observed in aging human and rodent skeletal muscle cells^38,39^ and has been attributed to defective or insufficient autophagy/mitophagy clearance of damaged organelles. It has been shown that the giant mitochondria have altered cristae, reduced membrane potential, and lack the ability to fuse with healthy organelles to buffer or repair damage through exchange or removal of materials. Although we showed that even giant mitochondria in *C. elegans* body wall muscle retain some membrane potential, those animals with sparse and enlarged mitochondria had lower overall basal respiration and spare capacity. Furthermore, they had reduced mitochondrial staining in non-muscle tissue. Together, these findings suggest that muscle mitochondrial morphology could be a surrogate indicator of whole-animal mitochondrial health.

*C. elegans* exercise conditioning, with or without food deprivation, protected against this aging-induced loss of networked mitochondrial morphology in nematodes, which is consistent with reports in mammalian systems. The tFD group was also somewhat protected compared to the control group, which may be a direct effect of mild food restriction or may be partly due to moderately increased physical activity level. Animals on food move slower than those without food, as the latter exhibit higher activity due to foraging behavior^40^. However, Laranjeiro *et al*. reported that the energy cost of swimming is much greater than that of crawling on agar^25^; therefore, while food-restricted animals may have higher physical activity than those on food, the amount of exercise should be much less than the swimming exercise group. Thus, effects in the tFD group are likely to be primarily due to a food deprivation response.

Higher basal respiration in exercised and tFD animals compared to the control, but not in animals exercised in food-containing media, suggests that transient food deprivation may result in more numerous or more active mitochondria in the whole organism. Furthermore, spare capacity was increased in exercised animals compared to control and tFD, but was not increased in the food exercise group. Thus, food deprivation contributes in some way to the effect of exercise on spare capacity. It has been reported that animals in liquid containing food swim less than those without food^41^, and thus food-swum animals may fail to achieve the full benefits afforded to the exercise group. Additionally, because these measurements were made in whole animals and not isolated mitochondria, differences in substrate availability cannot be ruled out as contributing factors to the oxygen consumption. However, it is clear that transient food deprivation along with exercise have dramatic effects on respiration in these animals. Future studies will be required to determine the mechanism by which these protections occur.

Proton leak was also increased on day 8 in exercised animals. Proton leak may reflect a higher level of fused mitochondria compared to the other groups^42^, or could represent an attempt to reduce levels of reactive oxygen species being produced by mitochondria in oxidized muscle^43,44^. In fact, Laranjeiro *et al*. reported higher muscle mitochondrial oxidation of redox-sensitive GFP following a single swim session^5^. Although that effect dissipated after 4 hours, repeated swim sessions may cause a recurrent or chronic prooxidant environment in muscle that could induce proton leak.

The lack of difference in mtDNA copy number among groups is somewhat surprising, given the improved mitochondrial morphology and networking in the body wall muscle and increased respiration. It is possible that there is a tissue-specific effect on mitochondria that is masked by the whole-animal determination of mitochondrial DNA copy number. There was also no difference in basal mitochondrial or nuclear DNA damage among the treatment groups; however, the basal level of nuclear DNA damage did increase between day 8 and day 12, which could indicate an aging-related increase in frequency of lesion formation or decline in repair, which has been documented^45^.

Although researchers have devoted considerable effort to studying the effects of exercise and environmental toxicants alone, very few studies have focused on the interaction between the two. Here, we have identified protections offered by exercise training against lethality from exposure to two common mitochondrial toxicants, arsenic and rotenone. We interpreted differences in lethality as differences in organismal toxicity; however, we cannot rule out differences in uptake between exercised, transiently food-deprived, and control animals at this point. Although we do not expect these interventions to cause differences in uptake, a mechanistic examination of these protections is needed in future studies.

Arsenic is a widespread drinking water contaminant to which millions of people worldwide are exposed every day at levels exceeding World Health Organization guidelines^46^. The mechanisms of arsenic toxicity are complex and not fully understood; however, mitochondria are an important target for arsenic toxicity, especially for the trivalent form (arsenite) used in this study^27,31,32^. The protection of exercised nematodes, with or without food, from arsenite toxicity demonstrated a dramatic and sustained protection compared to non-exercised controls. Swimming exercise in mice has recently been shown to attenuate arsenic-induced effects on long-term memory^47^. That study used a chronic (12-week) arsenic exposure paradigm; it is interesting to note that exercise appears to be protective in both chronic and acute arsenic exposures.

Rotenone is used worldwide as a pesticide. Interestingly, the protection against rotenone exposure that we found conferred by exercise (with or without food) against rotenone exposure was observed several days following cessation of exercise, indicating a persistence of protection after exercise conditioning. There are examples in the literature of exercise altering the toxic effects of chronic rotenone exposures in rodents. In rats, rotenone induces depression, motor dysfunction, alpha-synuclein expression, and dopaminergic neurodegeneration, all of which were attenuated by treadmill exercise performed during the same timeframe as the rotenone injections^48,49^. Furthermore, isolated arteries from young and old exercised mice had higher stress resistance after an *ex vivo* exposure to rotenone, compared to arteries from sedentary controls^50^.

The evidence for exercise intervention slowing the aging process is extensive^3,4^. In humans, exercise improves age-related decline in cardiovascular fitness, muscle function, frailty and weakness, neurological function, lung function, and metabolic fitness. Our data herein shows that in *C. elegans*, exercise, combined with transient food deprivation, not only protects mitochondria in muscle and in whole nematodes, but also results in a modest but significantly extended lifespan. Exercise also decreased the blue autofluorescence “death signal” in the exercised animals, which further supports an exercise-induced lifespan extension.

Two previous studies have reported lifespan extension after exercise in *C. elegans*. Chuang *et al*. used an electrotactic flow chamber to exercise nematodes for only 10 minutes per day for 6 days and reported a ~10% increase in lifespan^51^. Although that is a similar magnitude of lifespan extension to what we observed here, it is difficult to compare their study to this one because it is unclear how the electric field exposure may have impacted the worms. Chaudhari and Kipreos reported a 25–30% lifespan extension from swimming exercise (median lifespans of 15–19 days vs. 10–13 days for controls)^52^. In that case, swimming was carried out for 5–30 minutes per day until day 12. This is a much shorter daily swim session than we studied, but the regimen was carried out for much longer into life. These late-life swim sessions, however, are much more difficult and even dangerous for the worms; Chaudhari and Kipreos found it necessary to shorten the duration of swimming during the last days of exercise to prevent many animals from dying; our observations support a late-life challenge of extended swim (Laranjeiro, unpublished).

Based on the available literature on dietary restriction in *C. elegans*, it is not surprising that we did not see lifespan extension in the tFD group. Our protocol involved brief, transient periods of food deprivation that differ significantly from the lifespan-extending protocols in the literature. For example, longevity-conferring dietary restriction protocols include bacterial dilution, giving prolonged reduction in food intake^53–55^, impaired food intake in the long-lived *eat-2* mutant^53,56^, complete and sustained food deprivation following larval development^57,58^, and intermittent fasting involving long 24–48 hour sessions of dietary restriction^59,60^. These sustained dietary restriction and dietary deprivation sessions may be required to see lifespan extension benefits. Indeed, Lee *et al*. found that interrupting periods of dietary deprivation with *ad libitum* feeding negated the lifespan extension they observed^57^. The lack of lifespan extension in nematodes exercised in food-containing media suggests that the food deprivation is required for exercise to extend lifespan, but the food deprivation alone is not enough to extend lifespan.

For the reasons described above, a limitation of our studies is that interventions that increase exercise also decrease food intake, and interventions restricting food intake increase activity levels. Future studies are needed to fully disentangle the effects of food deprivation and exercise, and establish the mechanism(s) working in each case. In designing our study, we considered many options for control experiments. An ideal control would be an animal that could crawl relatively well but is unable to swim. Therefore, we tested several mutants with “fainter” phenotypes of this sort (*unc-79* (*e1068*)*, unc-79* (*ec1*)*, unc-80* (*e1272*)*, unc-80* (*e1069*))^61^, but discovered that although the fainter phenotype occurs immediately upon transferring the animals to liquid, it is not sustained – the animals begin to swim after a few minutes and have significant swimming activity during the 90 minute swim session (unpublished observations, Drs. Laranjeiro and Driscoll). We also tested other mutants reported to have a swimming defect, including *unc-18, unc-13, unc-104, unc-26*, and *ric-3*^62^. Unfortunately, these mutants either were paralyzed both on agar and in liquid, or moved fairly normally in both situations (unpublished observations, Drs. Laranjeiro and Driscoll). Thus, we were unable to identify a mutant that moved freely on agar but was paralyzed in liquid. Nonetheless, the exhaustive testing of control, transient food deprivation, exercise without food, and exercise with food has allowed us to begin to distinguish protections driven mainly by exercise from those driven by food deprivation. Overall, both swimming exercise and transient food deprivation provided benefits to the aging animals’ mitochondria. Ongoing and future studies will continue to characterize and distinguish these protections and the mechanisms underlying them.

To conclude, health benefits from regular exercise are myriad, and new protections from this simple and effective lifestyle intervention are still being discovered. However, the molecular mechanisms underlying these improvements are not well understood. To more adequately assess and improve the health of an aging population, and to potentially identify pharmacological targets, these mechanisms must be unveiled. It is estimated that the number of people aged >60 years will triple worldwide by 2050, with the greatest expansion being in the oldest population >85 years. At the same time, environmental exposures are increasing worldwide, causing ~16% of global deaths^13^, and new chemicals are being introduced to the environment before proper assessment of their long-term safety^63^. Therefore, identifying tractable strategies to improve personal health is critical to enhancing healthspan. *C. elegans* represent an inexpensive, efficient method to identify molecular mechanisms underlying exercise protections. We have shown herein that long-term exercise conditioning of adult nematodes confers protection from age-related decline in mitochondrial health and resistance against mitochondrial toxicants. Together, this represents a promising and exciting new model to answer critical new questions about exercise and toxicity.

## Methods

*C. elegans strains and culture*. The N2 Bristol wild-type strain and SJ4103 (zcls14 [myo-3::GFP(mit)] strain were obtained from the *Caenorhabditis* Genetics Center (CGC). Culture was on K-agar plates^64^ (Supplementary Method 1 for recipe) at 20°C unless otherwise noted.

### Exercise experiments

Synchronized populations of *C. elegans* were obtained by sodium hydroxide bleach treatment of gravid adults as previously described^65^, followed by overnight incubation of eggs and hatched larvae (16 hours) in complete K-medium^64^ (Supplementary Method 1 for recipe) on a rocker at 20 °C. Age-synchronized L1 (larval stage 1) nematodes were then maintained at 20 °C on 10 cm K-agar plates seeded with OP50 *E. coli* for 48 hours (approximately L4 stage) when exercise experiments were started. Exercise trainings were carried out at exactly 9:00 AM and 3:00 PM daily; timing was tightly regulated to control for circadian rhythms. For experiments, nematodes were washed off the plate with 10–12 mL of K-medium and allowed to gravity settle. The supernatant was removed and the animals were resuspended in 12 mL of K-medium to wash off residual bacteria and separate adults from larvae (when applicable). The worms were allowed to gravity settle again and the supernatant was removed. The adult worms were then transferred to an OP50-seeded K-agar plate (control group) or an unseeded K-agar plate lacking peptone (transient food deprivation, tFD, and exercise groups). The plates were dried for 10 minutes, and then 4 mL of complete K+ medium (Supplementary Method 1 for recipe) was added to the Exercise plate only, to induce swimming behavior. All plates were then transferred to the 20 °C incubator for 90 minutes. At the end of the 90-minute swim, nematodes were washed from the plate, gravity settled, and transferred back to K-agar plates seeded with OP50. This regimen was carried out for 6 days, ending on day 7 of life as measured from egg release.

Exercise conditioning took place during the entire reproductive period for hermaphrodites (Fig. 1b), which lay eggs beginning at approximately 2.5 days and lay an average of 4.6 days (meaning day 7 should be the last day to lay eggs, and eggs will be few compared to earlier reproductive days)^66^. However, as our populations of animals are normally 1500 per plate, and assuming that males constitute 0.1–0.2% of the population, we would expect 1–3 males per plate. Mated hermaphrodites lay eggs much longer, an average of 8.8 days, which means that some hermaphrodites may continue to lay fertile eggs until day 11. Therefore, animals were transferred off of larvae every day between day 8 and day 12 endpoints to avoid confounding effects from inclusion of offspring in measurements.

### DNA damage and copy number experiments

As a measure of nematode genomic integrity and as an indirect measure of mitochondrial biogenesis, we determined DNA damage and copy number for nuclear and mitochondrial genomes. For experiments, mitochondrial and nuclear DNA lesions and copy number were determined as previously described^29^, by amplification of a long >10 kb fragment of DNA normalized to a real-time qPCR quantification of copy number (compared to a standard curve). The relative amplification of intact long fragments indicates the relative integrity of each genome. Briefly, on day 8 and day 12, six animals per technical replicate were picked into 90 µL 1Xworm lysis buffer (25 mM Tricine, pH 8; 80 mM potassium acetate; 11% w/v glycerol; 2.25% v/v DMSO, 1 mg/mL proteinase K in nuclease-free water) and frozen at −80 °C for at least 20 minutes. Following, the animals were lysed at 65 °C for 1 hour, and the lysate was used as a template in long QPCR (DNA damage) and real-time PCR (copy number) experiments. For long QPCR experiments, LongAmp *Taq* DNA Polymerase (New England Biosciences, Ipswich, MA) was used with 5 µL of lysate for the template. For real-time PCR experiments, Power Sybr Green PCR Master Mix (ThermoFisher Scientific, Waltham, MA) was used with 2 µL of lysate for the template. C_T_ values were converted to copy number using a standard curve with the pCR 2.1 plasmid containing the species-specific mitochondrial *nduo-1* gene fragment for mtDNA or the lysate from *glp-1* young adult worms with a fixed cell/nuclear copy number count. Primer sequences, amplicon size, and annealing temperatures are included in Supplementary Data Table S1.

### Mitochondrial morphology experiments

For measurement of mitochondrial morphology, the strain SJ4103 expressing mitochondrial matrix-targeted GFP in body wall muscle was used. For imaging, worms were picked onto a 2% agarose pad containing 10 mM sodium azide and covered with a coverslip. Confocal imaging was performed on a Zeiss LSM 510 confocal microscope with a 40× objective, scanning with a 488 nm laser at 1 mW power with 100 ms exposure. For each animal, the posterior portion of the animal was imaged (just anterior to the tail). Stacks were taken beginning at −15 µm relative Z at 1 µm intervals, 10 images total. The image(s) in the stack containing the muscle mitochondrial staining was resaved. Images were then scored blindly using a software program written in-house, which allows the user to define a scoring system. Following, the software presents each image in a random order to the user and allows the user to the select the score from a drop-down menu. Some images are presented multiple times for quality control purposes. In the end, scores are unblinded and presented to the user as compiled numbers for each group.

Follow-up experiments were performed with MitoTracker Red CMX-Ros dye, using a previously published method^67^. Briefly, 200 12-day old SJ4103 animals (harboring mitochondrial-targeted GFP in body wall muscle) were washed off of plates in M9 solution and transferred to a 0.5 mL amber tube. Animals were allowed to settle and the liquid was aspirated; following, 0.4 mL of M9 was added to wash the worms. This process was repeated a total of 3 times. Following, the M9 was aspirated and replaced with 200 µL of dye in a final concentration of 5 µM (0.5% DMSO) and a density of 1 worm/µL. The worms were placed on a rotator at 20 °C for 1 hour. Following, the animals were loaded onto a 2% agarose pad containing sodium azide and covered with a coverslip. Confocal imaging was performed on a Zeiss LSM 510 confocal microscope with a 40× objective, scanning with a 488 nm (GFP) and 561 nm (Mitotracker Red) laser, each at 1 mW power with 100 ms exposure.

### Respiration experiments using the Seahorse XCFa

Respiration experiments were performed as previously described for L4 animals^68^, with some minor adjustments due to the age of the animals. Briefly, worms were washed off of the plates into 10 mL K-medium and allowed to gravity settle. The supernatant was aspirated and the animals were resuspended in 12 mL K-medium. This washing process was repeated once more, and the worms were finally resuspended in 10 mL K-medium and placed at 20 °C on a rocker for 20 minutes to allow them to clear their guts of bacteria. Following, animals were counted and the volume was adjusted to generate a final concentration of 1 worm per 2 µL. Twenty-five animals were then added to each well in the 24-well plate in 50 µL K-medium, leaving 2–4 wells for blanks. An additional 100 µL K-medium was added to each well, followed by 375 µL of EPA water^68^ (a lower-saline solution to ensure solubility of drugs, Supplementary Method 1 for recipe). Preliminary experiments revealed that older animals require higher salt concentrations than are present in EPA water to maintain their integrity (unpublished observations), so the addition of K-medium is critical for older adult animals. Basal respiration was measured first in every case, followed by injection of FCCP (Carbonyl cyanide-p-trifluoromethoxyphenylhydrazone; to uncouple mitochondria) or DCCD (dicyclohexylcarbodiimide; to inhibit ATP synthesis) followed by sodium azide (to completely inhibit mitochondrial respiration). At the end of the respiratory measurements, the animals were manually counted (we find this is critical due to the small number of animals per well) and respiration was adjusted to number of animals and to average volume of the group.

### Lethality experiments with arsenite and rotenone

For lethality experiments, nematodes (10 per replicate in a 96 well plate) were incubated with increasing concentrations of the chemical (sodium arsenite or rotenone) and UV-inactivated UVRA bacteria (to reduce bacterial transformation of the chemical, as previously described^69^) in complete K-medium for 24 hours. After 24 hours, survival was assessed by a harsh touch with a platinum wire. Animals that failed to respond to three harsh touches were considered to be dead. Each experiment was performed in duplicate technical replicates for each exposure dose, and three independent biological replicates.

### Lifespan experiments

Lifespan experiments were initiated after the last day of exercise. Therefore, any deaths that occurred prior to day 8 were not considered. On day 8, 50 animals were transferred to a 6 cm seeded plate. The animals were transferred to new plates as necessary to avoid food depletion, and survival was assessed by a light tap with a platinum wire every 1–2 days. The experiment was repeated for three independent biological replicates.

### Assessing autofluorescence in 8- and 12-day old animals

For measuring autofluorescence, worms were mounted on a glass microscope slide on a 2% agarose pad and immobilized with sodium azide. As a beginning step and to identify proper placement of worms within the frame, transmitted light images were obtained followed by 3 autofluorescence images. Autofluorescence was captured for blue (DAPI channel, Ex. 357 nm/Em. 447 nm, 80% light, 120 ms exposure), green (GFP channel, Ex. 470 nm/Em. 510 nm, 90% light, 250 ms exposure), and red (Texas Red channel, Ex. 585 nm/Em. 624 nm, 100% light, 1.0 s exposure) autofluorescence on an AMG EVOS FL digital inverted microscope. Whole-nematode fluorescence was then analyzed using ImageJ using the following procedure:

For each individual nematode, the transmitted light image was digitally stacked with the 3 fluorescence images. An outline of the entire organism was selected in the transmitted light image using the ImageJ “Wand tool” with tolerance set at 60 and mode set as Legacy. This selection was used as the area of interest throughout the paired autofluorescence images. Each of the three fluorescent images were then analyzed using the ImageJ “Measure” function, outputting multiple endpoints, such as area, perimeter, and mean intensity. Data presented (Fig. 6b and Supplementary Fig. S6) represent mean intensity (integrated density divided by area, to account for size difference between treatment groups).

### Swimming exercise in food-containing media

Initially, swimming exercise experiments were performed with two different concentrations of food, with OD_600_ values of 0.4 and 1.2; however, after the fifth day of exercise, animals exercised in the higher food concentration began to die following the exercise regimen, possibly due to oxygen depletion. Therefore, only the lower concentration of food was used for subsequent experiments. For experiments, OP50 was inoculated into an overnight culture and grown to saturation. Following, the saturated bacteria was kept at 4 °C until use. For each exercise experiment, the saturated bacterial culture was pelleted and resuspended in complete K+ medium at 5-fold (OD_600_ = 0.4) or 5/3-fold (OD_600_ = 1.3) dilution of the saturated culture. The adult worms were then transferred to an OP50-seeded K-agar plate (control group) or an unseeded K-agar plate lacking peptone with 4 mL of bacteria in complete K+ medium (food exercise group). The plates were then transferred to the 20 °C incubator for 90 minutes. At the end of the 90-minute swim, nematodes were washed from the plate, gravity settled, and transferred back to K-agar plates seeded with OP50. This regimen was carried out for 6 days, as with the previous exercise experiments.

### Statistical analyses

All statistical analyses were performed in GraphPad Prism 7.0. For most experiments (including DNA damage, DNA copy number, respiration, and autofluorescence), a two-way ANOVA considering age and treatment group was performed, followed by a Bonferroni test to compare individual groups. For lethality experiments, a two-way ANOVA considering treatment group and dose was used as well as a global pairwise comparison of the nonlinear fits to the LC_50_ equation using the extra sum-of-squares F test. For lifespan, significance of differences between survival curves as well as median lifespans were analyzed using the Mantel-Cox log-rank test, first globally to test for a significant difference among all three groups followed by pairwise comparison for differences between individual groups. Finally, for mitochondrial morphology, a Chi-squared test was used to determine significance of the distribution differences between all three groups globally. Following, pairwise comparisons were made using the Chi-squared test between each group to determine significant differences between groups, adjusting the significance level to α = 0.05/3 = 0.017 (Bonferroni correction) to control for multiple comparisons^70^.

### Availability of data and material

The datasets used and/or analyzed during the current study are available from the corresponding author on reasonable request.

## Electronic supplementary material

Supplementary Information
