# Supplementary material for: Swimming Exercise and Transient Food Deprivation in Caenorhabditis elegans Promote Mitochondrial Maintenance and Protect Against Chemical-Induced Mitotoxicity
Source: Sci Rep. 2018 May 29;8:8359. doi: 10.1038/s41598-018-26552-9 (PMC5974391; doi:10.1038/s41598-018-26552-9)
Supplement: Supplementary file 1 — Supplementary Information [file 41598_2018_26552_MOESM1_ESM.docx]

**SUPPLEMENTARY INFORMATION**

**Swimming Exercise and Transient Food Deprivation in *Caenorhabditis elegans* Promote Mitochondrial Maintenance and Protect Against Chemical-Induced Mitotoxicity**

Jessica H. Hartman*^1^, Latasha L. Smith^2^, Kacy L. Gordon^3^, Ricardo Laranjeiro^4^, Monica Driscoll^4^, David R. Sherwood^3^, Joel N. Meyer^1^

*^1^Nicholas School of the Environment, Duke University, Durham, NC United States*

*^2^Department of Pharmacology and Cancer Biology, Duke University, Durham, NC United States*

*^3^Department of Biology, Duke University, Durham, NC United States*

*^4^Department of Molecular Biology and Biochemistry, Nelson Biological Laboratories, Rutgers, The State University of New Jersey, Piscataway, NJ United States*

*Corresponding Author:

Jessica H. Hartman, Nicholas School of the Environment, Duke University, 9 Circuit Drive, Durham, NC, USA; Telephone: 919.613.8027; Fax: 919.668.1799; Email: [jessica.h.hartman@duke.edu](mailto:jessica.h.hartman@duke.edu)

**TABLE OF CONTENTS**

**Supplementary Fig. S1. Transient food deprivation reduces worm size..… 3**

**Supplementary Fig. S2. ATP-linked OCR unchanged by exercise………….. 4**

**Supplementary Fig. S3. Individual traces for arsenic lethality experiment .. 5**

**Supplementary Fig. S4. Individual traces for rotenone lethality experiment 6**

**Supplementary Fig. S3. Individual lifespan traces …………………………… 7**

**Supplementary Fig. S4. Red and green autofluorescence …………………… 8**

**Supplementary Table S1. QPCR primers and analysis details ……………… 9**

**Supplementary Table S2. Arsenite LC_50_ values after exercise in food …...... 10**

**Supplementary Table S3. Rotenone LC_50_ values after exercise in food ……. 10**

**Supplementary Method 1. Recipes for K-agar, K-medium, EPA water …… 11**

**Supplementary References ………………………………………………………… 12**

**Figure S1. Transient food deprivation causes persistent decrease in adult worm size.** Representative data shown are compiled from two biological replicates (n>35 animals per replicate). For imaging, worms were transferred to unseeded agar plates on day 8 and imaged using a Nikon SMZ 1500 stereomicroscope at 10X magnification with a Nikon DXM 1200 camera and Nikon NIS-Elements 3.20.01 software. Worm length, width, and volume were then measured using the freely available FIJI (ImageJ 2) plugin WormSizer*^1^*, developed by the Baugh lab. Asterisks indicate significance: ***, p<0.0001; n.s., not significant as determined by one-way ANOVA followed by a Tukey test.

**Figure S2. ATP-linked OCR unchanged by exercise.** For experiments, nematodes were washed off plates, rinsed, and allowed to clear their guts, then loaded into 24-well Seahorse plates, 25 nematodes per well, for measurement of oxygen consumption rate (OCR). Basal OCR was measured along with OCR following injection of FCCP, DCCD, or sodium azide. These measurements were then normalized per nematode and per volume and used to calculate ATP-linked respiration (basal OCR - DCCD response). Statistical significance was assessed by a two-way ANOVA followed by a Bonferroni posttest comparing all groups to each other. Data represent the mean and standard error from 3 biological replicates.

**
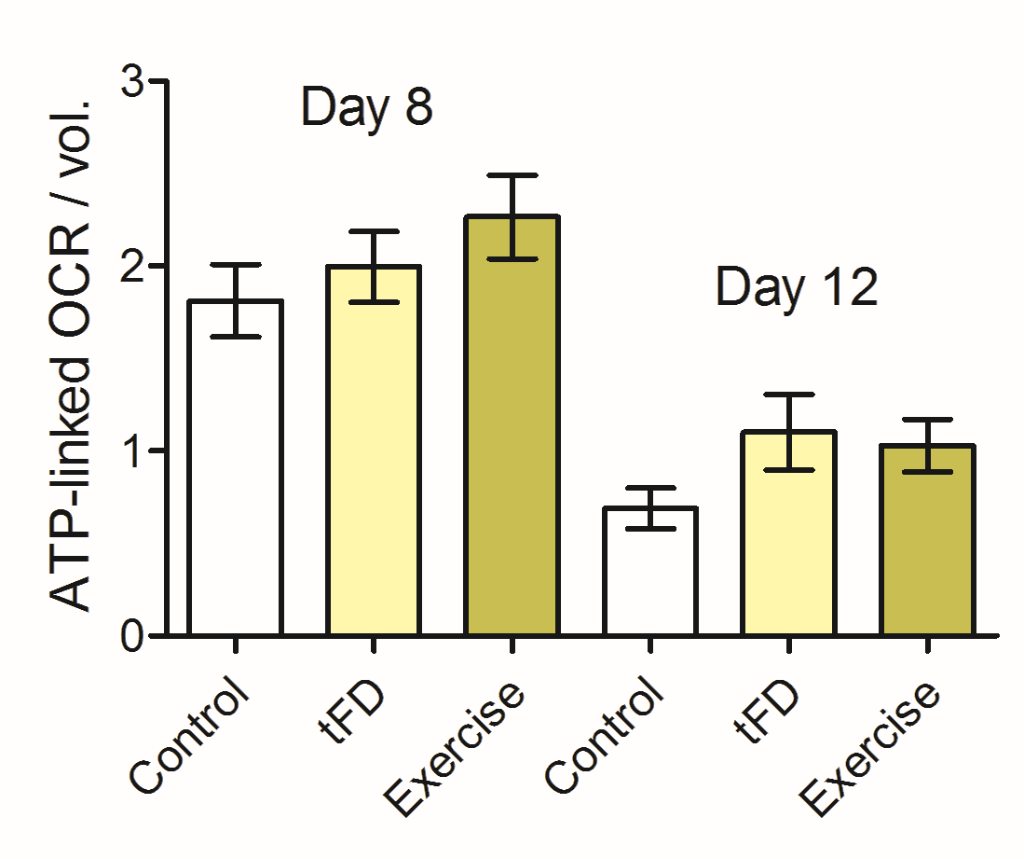
**

**Figure S3.** **Individual traces for arsenic lethality.** Each panel represents a lethality experiment from an independent biological replicate with n=20 animals per group (10 animals in each of 2 wells). Panels A and B are from one biological replicate and represent lethality on days 8 and 12, respectively. Likewise, panels C and D are from the second replicate and panels E and F are from the third biological replicate. Compiled results with statistical analysis are included in the body of the manuscript.


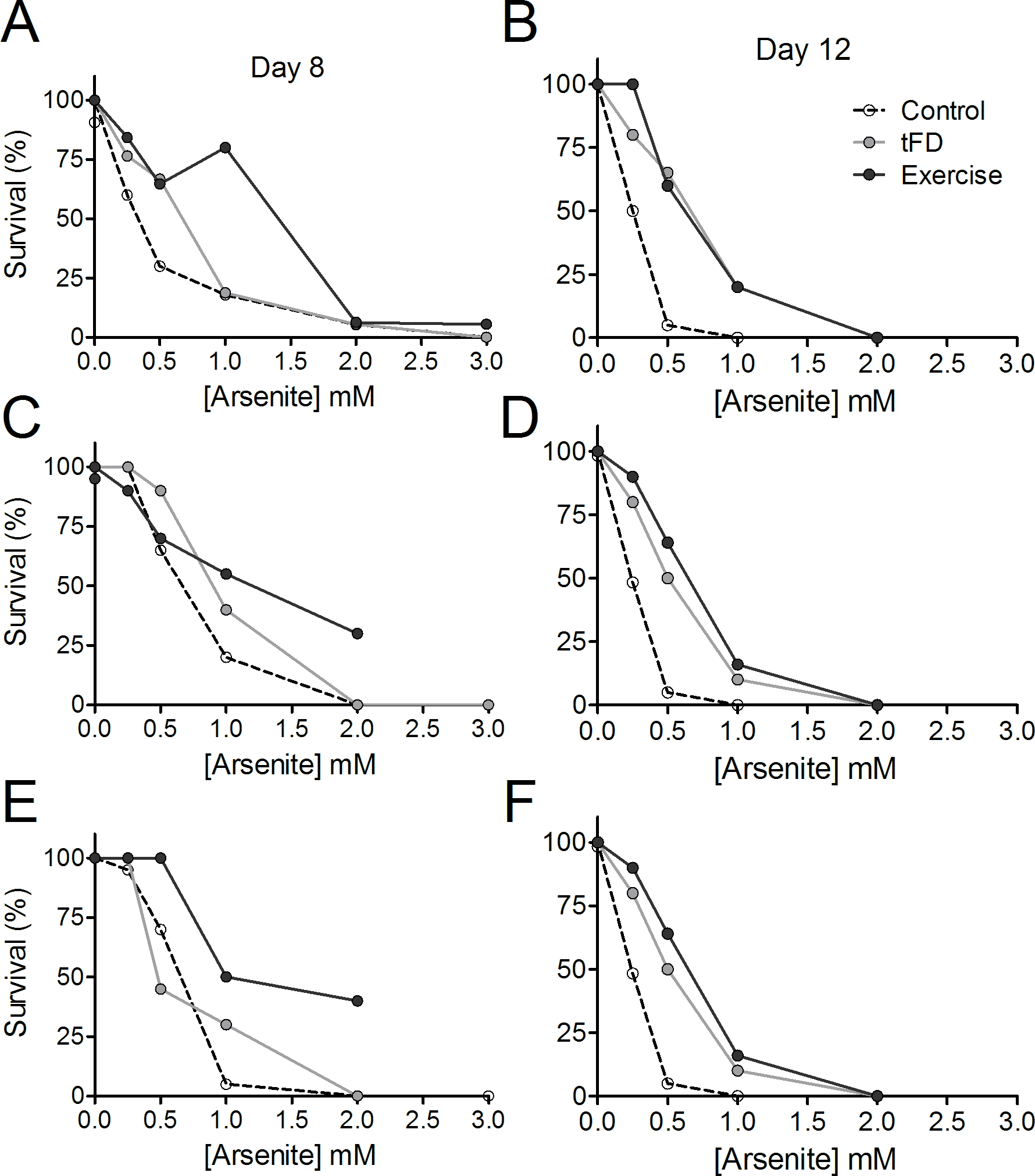


**Figure S4. Individual traces for rotenone lethality.** Each panel represents a lethality experiment from an independent biological replicate with n=20 animals per group (10 animals in each of 2 wells). Panels A and B are from one biological replicate and represent lethality on days 8 and 12, respectively. Likewise, panels C and D are from the second replicate and panels E and F are from the third biological replicate. Compiled results and statistical analyses are included in the body of the manuscript.

**
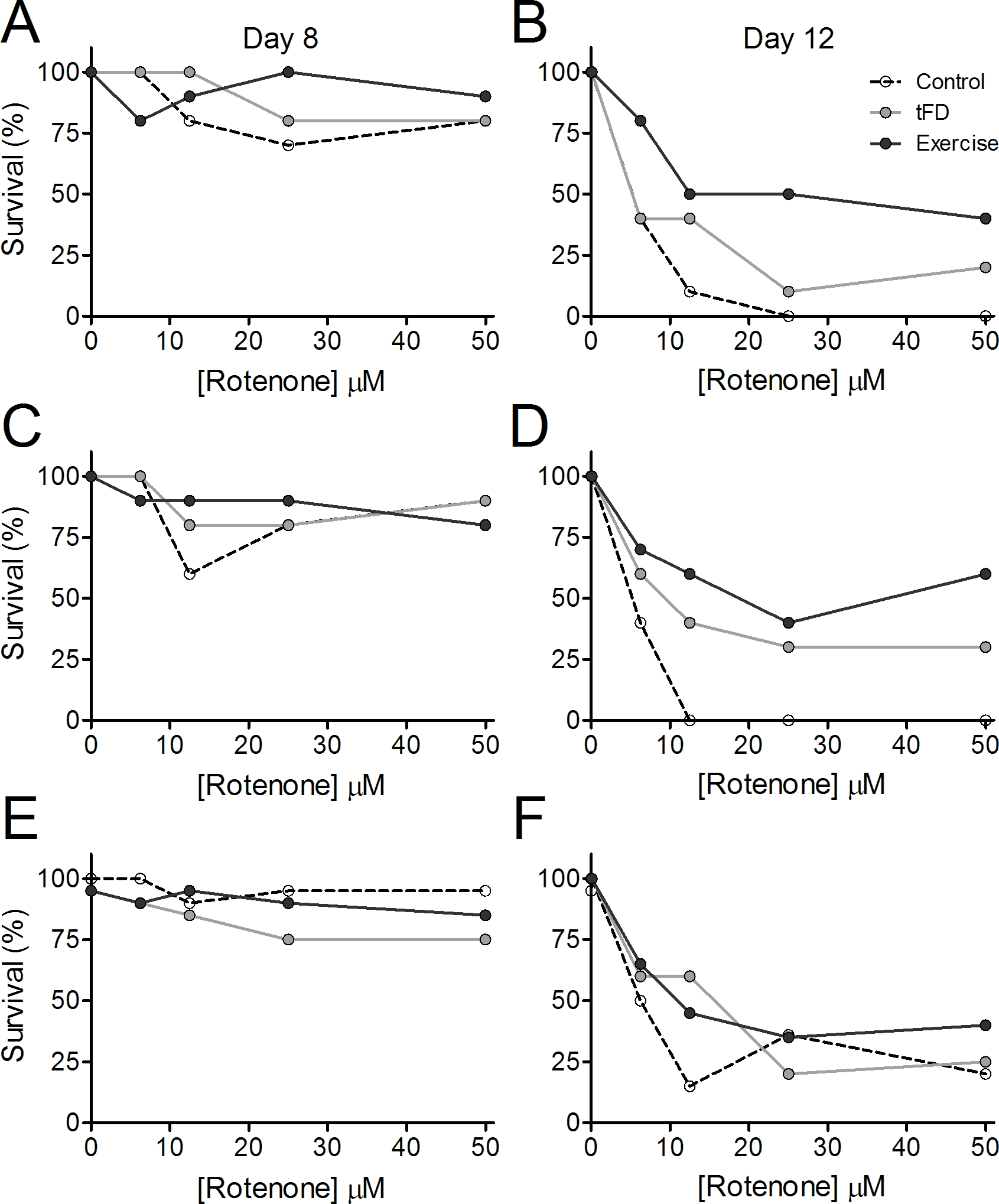
**

**Figure S5. Individual traces for lifespan experiment.** Each panel represents a lifespan experiment from an independent biological replicate with n=50 animals per group. Statistical significance and p-values displayed on panels A-C were determined by a global comparison of all three groups using the Mantel-Cox (log-rank) test. Median lifespan and pairwise comparisons are shown in Panel D using the Mantel-Cox test.

**
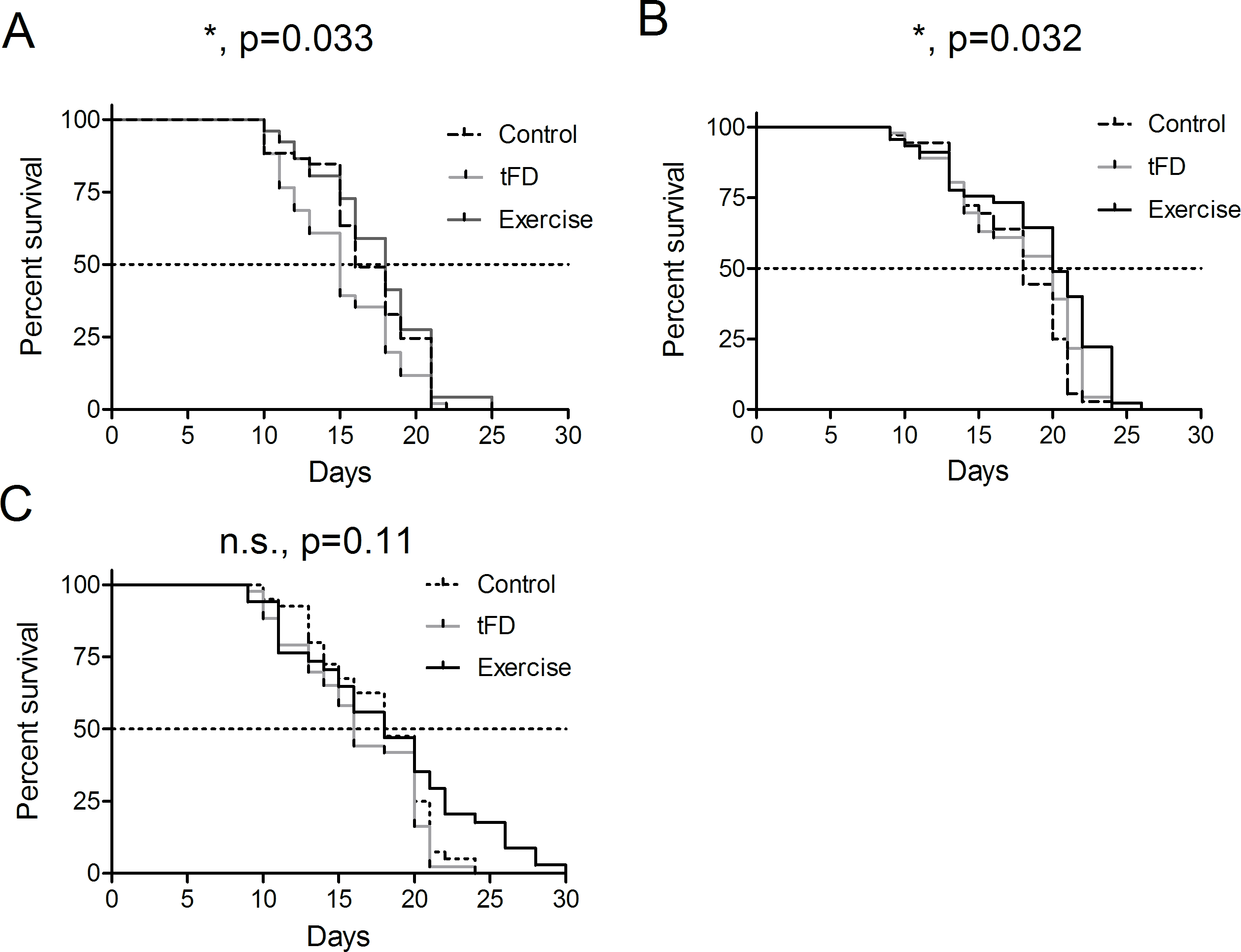
**

D

| **Replicate** | **Group** | **Mean Lifespan, days (SD)** | **Median Lifespan, days** | **Significance?** | |
| --- | --- | --- | --- | --- | --- |
|  |  |  |  | **vs. Control** | **vs. tDR** |
| 1  (Panel A) | Control | 17 (3.5) | 16 | − | − |
|  | tFD | 15 (3.6) | 15 | *, p=0.037 | − |
|  | Exercise | 17 (3.6) | 18 | n.s. | **, p=0.0031 |
| 2  (Panel B) | Control | 18 (3.7) | 18 | − | − |
|  | tFD | 18 (4.3) | 20 | n.s. | − |
|  | Exercise | 19 (4.7) | 20 | **, p=0.0030 | *, p=0.032 |
| 3  (Panel C) | Control | 17 (3.8) | 18 | − | − |
|  | tFD | 16 (4.2) | 16 | n.s. | − |
|  | Exercise | 18 (6.0) | 18 | n.s. | *, p=0.023 |

**Figure S6. Red and green autofluorescence in control and exercised animals.** Although the pigment responsible for blue autofluorescence has been identified and linked to a “death signal”, the autofluorescent material for red and green fluorescence is still unclear. Shown below is red and green autofluorescence normalized to worm area. Statistical significance was assessed by a one-way ANOVA followed by a Tukey posttest comparing all groups to each other. Significance is indicated by asterisks: *, p<0.05; **, p<0.01.

**
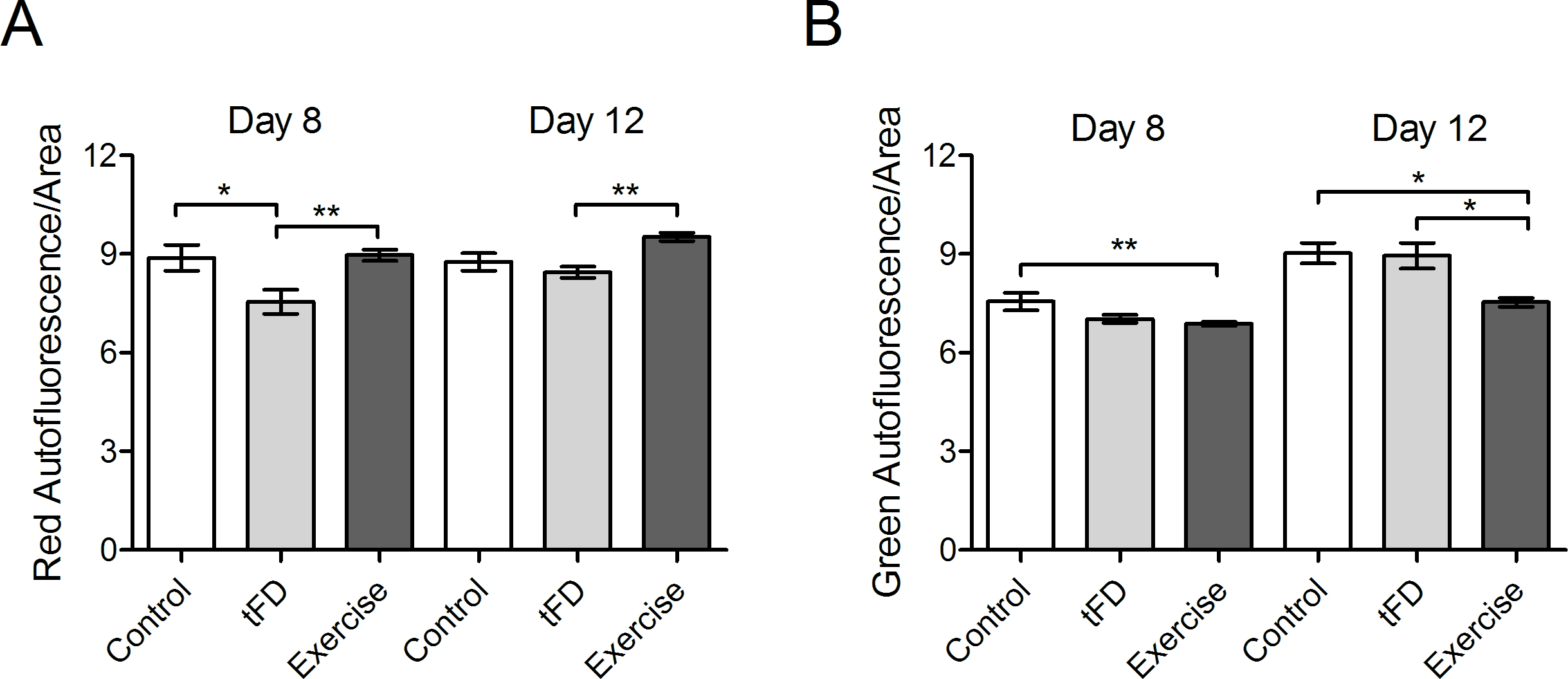
**

**Table S1. QPCR DNA copy number and damage assay.** Determination of DNA copy number and size for mitochondrial and nuclear genomes was carried out as previously described*^2^*. Primer sequences, annealing temperatures, and other details are provided below.

***Long QPCR amplification for DNA damage***

| **Genome** | **Forward Primer Seq** | **Reverse Primer Seq** | **Annealing Temp. (°C)** | **Cycle Number** | **Amplicon Size (kbp)** |
| --- | --- | --- | --- | --- | --- |
| **Mitochondrial** | 5’-CCA TCA ATT GCC CAA AGG GGA GT-3’ | 5’-TGT CCT CAA GGC TAC CAC CTT CTT CA-3’ | **64** | **25** | **10.9** |
| **Nuclear** | 5’-TGG CTG GAA CGA ACC GAA CCA T-3’ | 5’-GGC GGT TGT GGA GTG TGG GAA G-3’ | **64** | **29** | **9.3** |

***Real-time QPCR analysis of copy number***

| **Genome** | **Forward Primer Seq** | **Reverse Primer Seq** | **Annealing Temp. (°C)** | **Gene** | **Amplicon Size (bp)** |
| --- | --- | --- | --- | --- | --- |
| **Mitochondrial** | 5’-AGC GTC ATT TAT TGG GAA GAA GAC-3’ | 5’- AAG CTT GTG CTA ATC CCA TAA ATG T -3’ | **60** | **nd-1** | **75** |
| **Nuclear** | 5’-GCC GAC TGG AAG AAC TTG TC-3’ | 5’-GCG GAG ATC ACC TTC CAG TA-3’ | **60** | **cox-4** | **164** |

**Table S2. LC_50_ values after 24-hour exposure to sodium arsenite.** Animals were subjected to twice-daily 90 min swim exercise in media containing bacterial food beginning at young adult and continuing for 6 days. Lethality was tested on day 8 (the day after ceasing swim exercise) and on day 12. Significance was determined by pairwise comparison of the nonlinear LC_50_ fits using the extra sum-of-squares F test.

| **Group** | **LC_50_, mM (standard error)** | **Significance?** |
| --- | --- | --- |
|  |  | **vs. Control** |
| *Day 8* |  |  |
| Control | 0.9 (0.24) | − |
| Food Exercise | 4.6 (1.2) | ***, p = 0.0002 |
| *Day 12* |  |  |
| Control | 0.32 (0.075) | − |
| Food Exercise | 0.67 (0.11) | ***, p = 0.0003 |

**Table S3. LC_50_ values after 24-hour exposure to rotenone.** Animals were subjected to twice-daily 90 min swim exercise in food beginning at young adult and continuing for 6 days. Lethality was tested only on day 12, due to high resistance to rotenone on day 8. Significance was determined by pairwise comparison of the nonlinear LC_50_ fits using the extra sum-of-squares F test.

| **Group** | **LC_50_, µM (standard error)** | **Significance?** |
| --- | --- | --- |
|  |  | **vs. Control** |
| *Day 12* |  |  |
| Control | 7.2 (1.4) | − |
| Food Exercise | 21 (1.4) | *, p=0.0362 |

**Method 1. Recipes for K-agar plates and K-medium**

**K-agar Plates:**

Weigh 2.36 g KCl, 3 g NaCl, 2.5 g peptone, and 20 g agar into a 2 L flask. Add 1 L of distilled water, and autoclave (30 min liquid cycle). After cooling to 55°C, add 1 mL 1M CaCl_2_, 1 mL 1M MgSO_4_, 1 mL 10 mg/mL cholesterol, and 5 mL 1.25 mg/mL nystatin (optional). Pour plates and seed within 24 hours with bacteria.

**K-medium:**

Weigh 2.36 g KCl and 3 g NaCl per L of K-agar into a flask. Add distilled water and autoclave (30 min liquid cycle).

**K+ medium:**

To 50 mL of K-medium, add 150 µL 1M CaCl_2_, 150 µL MgSO_4_, and 25 µL 10 mg/mL cholesterol**.** Mix well and use fresh.

**EPA water:**

In 1 L distilled water: 96 mg NaHCO_3_, 60 mg MgSO_4_ (heptahydrate), 60 mg CaSO_4_ (dihydrate), 8 mg KCl. Mix well and filter sterilize if desired.

**Supplementary References**

[1] Moore, B. T., Jordan, J. M., and Baugh, L. R. (2013) WormSizer: high-throughput analysis of nematode size and shape, *PLoS One* *8*, e57142.

[2] Gonzalez-Hunt, C. P., Rooney, J. P., Ryde, I. T., Anbalagan, C., Joglekar, R., and Meyer, J. N. (2016) PCR-Based Analysis of Mitochondrial DNA Copy Number, Mitochondrial DNA Damage, and Nuclear DNA Damage, *Curr Protoc Toxicol* *67*, 20.11.21-20.11.25.
